# School-Level Socioeconomic Status and Nutrient Content of Outdoor Food/Beverage Advertisements

**DOI:** 10.3390/ijerph20186730

**Published:** 2023-09-07

**Authors:** Phoebe R. Ruggles, Jacob E. Thomas, Natalie S. Poulos, Keryn E. Pasch

**Affiliations:** 1 Department of Kinesiology & Health Education, University of Texas at Austin, Austin, TX 78712, USA; 2 School of Community and Rural Health, Heath Science Center, The University of Texas at Tyler, Tyler, TX 78708, USA; 3 Department of Nutritional Sciences, College of Natural Sciences, The University of Texas at Austin, Austin, TX 78712, USA

**Keywords:** food/beverage advertising, marketing, SES, schools, youth, outdoor advertising

## Abstract

We examined if areas around schools with more students of lower socioeconomic status (SES) have more total food/beverage advertisements and/or more advertisements with poorer nutritional content as compared to areas around schools with fewer students with lower SES. All outdoor food/beverage advertisements within a half-mile radius of 47 middle and high schools in the United States were objectively documented in 2012 and coded for nutritional content. The total number of advertisements and the macronutrient and micronutrient contents (total calories, fat (g), protein (g), carbohydrate (g), sugar (g), and sodium (mg)) of food and beverage items depicted in the advertisements were calculated. In total, 9132 unique advertisements were recorded, with 3153 ads displaying food and beverages that could be coded for nutrient content. Schools located in areas of lower SES (≥60% students receiving free/reduced-price lunch) had significantly more advertisements displaying food and beverages that could be coded for nutrient content (z = 2.01, *p* = 0.04), as well as advertisements that contained more sodium (z = 2.20, *p* = 0.03), as compared to schools located in areas of higher SES. There were no differences in calorie, fat, protein, carbohydrate, or sugar content. Policies to reduce the prevalence of outdoor food and beverage advertising are warranted.

## 1. Introduction

Childhood obesity is a pressing public health concern, affecting 19.3 percent of youth aged 2 to 19 years [1]. Childhood obesity is a complex issue and is associated with a variety of behavioral, environmental, psychosocial, and genetic factors [2]. Of the many factors associated with the development of overweight and obesity among youth, dietary behaviors are particularly influential in weight status [3]. Developing obesity is problematic to health, as people who experience obesity are at a greater risk for chronic diseases, such as Type II diabetes, high blood pressure, and asthma [4]. Those who experience obesity in their youth are more likely to remain obese as adults, thereby prolonging the negative health outcomes associated with having obesity [4].

The prevalence of obesity varies by sociodemographic factors. Those who experience obesity are disproportionately represented in under-resourced communities, with 18.9% of children in families in the lowest income bracket (<130% of the federal poverty line (FPL)) experiencing obesity, compared to 10.9% in the highest income bracket (>350% FPL) [5]. Families in areas with limited resources also face exposure to more environmental contributors that increase risk for having obesity [6]. For example, youth who live in communities with lower incomes [7,8,9,10,11] are more likely to be exposed to unhealthy food and beverage advertisements and live in areas with greater access to unhealthy foods and beverages [12]. Additionally, Black and Hispanic/Latinx youth have a higher prevalence of obesity compared to other racial/ethnic groups [1], and predominately Black and Hispanic/Latinx communities have higher densities of outdoor food and beverage advertisements compared to predominantly white communities [7]. These disparities, particularly in food and beverage advertising density, may suggest that communities are targeted with advertisements based on sociodemographic makeup.

Obesogenic environments, in which characteristics of the environment create barriers to maintaining a healthy weight [13], are thought to be some of the most significant modifiable contributors to the high prevalence of obesity [14,15]. Specifically, the prevalence of inexpensive, highly palatable, convenient food and beverage products, in combination with persuasive and omnipresent food and beverage marketing, are thought to be key, directly modifiable, drivers of obesity [14,15]. Youth are particularly vulnerable to marketing within obesogenic food environments, such as convenience stores or quick-serve fast food restaurants, due to their purchasing power and susceptibility to persuasive marketing [16,17]. Many convenience stores and fast-food restaurants epitomize the key components of an obesogenic food environment, as they tend to stock primarily non-perishable, energy-dense, highly processed snack foods and beverages. Furthermore, convenience stores and fast food restaurants tend to have extensive advertising for these products inside and outside of the store [18]. When youth live and attend school in close proximity to convenience stores, they are more likely to engage in unhealthy eating behaviors such as frequently purchasing snacks and other unhealthy food [19]. Furthermore, the most common foods youth purchase at convenience stores and quick-serve restaurants are calorically dense and low in nutritional value [20]. Thus, food environments influence food preferences and purchases in youth.

Outdoor food and beverage advertising is a key aspect of the food environment, particularly around schools [21,22,23,24,25]. Outdoor advertisements are promotional materials that prompt eating or drinking that are located outside, such as billboards, window posters, outdoor banners, or A-frame sandwich boards. Food and beverage companies expend millions of dollars on outdoor advertising each year. Data from the Rudd Center for Food Policy and Obesity show that in 2019, fast food companies spent $185 million on outdoor advertising [26], and in 2018, beverage companies spent $46 million [27]. Studies report that outdoor food and beverage advertisements overwhelmingly promote foods that are broadly considered to be less healthy [28,29,30,31,32,33]. Several systematic reviews and meta-analyses have found that youth are more likely to select advertised products over non-advertised products [34] and that youth tend to consume more food after exposure to advertisements [35,36]. Knowing the effects of advertising exposure on children’s food and beverage product preferences and consumption patterns, the prevalence of unhealthy food and beverage product advertising is concerning.

The outdoor food and beverage marketing environment near schools is well studied [9,24,29,30,31], but little is known about two important aspects of this environment: the specific nutrient content of the advertised products and whether the nutrient contents of the products vary by neighborhood income. Understanding the specific nutrient contents of advertised products, as opposed to broader categorizations (i.e., “unhealthy” vs “healthy), is important because the health implications of overconsumption vary by the type of nutrient. For example, overconsumption of added sugars can increase the risk of developing dental caries [37], while overconsumption of sodium does not increase this risk. Past research has examined the nutrient content of foods and beverages advertised during children’s television programs, finding that the products were high in added sugar [38,39,40] and sodium [39,40], both of which are nutrients that should be limited. However, there is a lack of research examining the nutrient content of outdoor food and beverage advertisements. Adams, Ganiti, and White evaluated the nutrient contents of outdoor food and beverage advertisements by socioeconomic status of the communities [8]. This study did not find any evidence of an association between socioeconomic status and nutrient content of advertised food items, although they did find that the advertised items were high in fat and sodium across all areas. However, the sample of food advertisements was small (n = 211) and limited to one city in the United Kingdom [8]. Further evidence is needed to inform our understanding of the outdoor food and beverage advertising environment in other geographic locations as well as near child-serving institutions.

Knowing that the food and beverage products advertised on child-oriented television programs are nutrient-deficient [38,39,40] and that most of the food and beverage items featured in outdoor advertisements are considered less healthy [21,28,29,32] the nutrient contents of products in outdoor food and beverage advertisements are likely deficient. Factors such as the sociodemographic composition of neighborhoods may be related to the prevalence and type of advertisements in local food environments [41]. The prevalence of food and beverage products and the disproportionate density of unhealthy food and beverage advertising in communities with limited resources and around child-serving institutions suggests that children from communities with limited resources could be exposed to more outdoor food and beverage advertisements for nutrient-deficient products than children from higher-income communities. Given the disparities in the prevalence of children who experience overweight and obesity by economic status, along with the impact marketing exposure has on dietary behaviors, it is crucial to understand if the nutrient content of products advertised near schools varies by school-level socioeconomic status. This will aid in understanding how food environments may be associated with diet-related health disparities. The present pilot study explores this gap in the literature by comparing the total number of food and beverage advertisements, or prevalence, around both middle and high schools as well as the nutrient content depicted in these advertisements by school-level socioeconomic status (SES) (as measured by the percentage of students who qualify for free or reduced-priced lunch (FRPL)). Because this was an exploratory pilot study, we did not propose a specific hypothesis but instead had two primary objectives. Our first objective was to examine the prevalence of outdoor food and beverage advertisements and if this prevalence differed significantly in areas around schools by school-level SES (e.g., schools located in areas of lower or higher SES as defined by FRPL). Our second objective was to assess if the nutrient content of the food and beverage items displayed in these outdoor advertisements differed significantly by school-level SES.

## 2. Methods

The data used in this exploratory pilot study were collected for the Outdoor Measuring and Evaluating Determinants and Influences of Advertising (MEDIA) Study [42,43] between February and July 2012. The purpose of the Outdoor MEDIA study was to document and describe all of the outdoor food and beverage, tobacco, and alcohol marketing materials within a half-mile radius of 34 middle schools (grades 6–8), 13 high schools (grades 9–12), and 9 hospitals in and around Austin, Texas. Hospitals were included in the data collection phase to act as a comparator for schools, as they are also community-serving institutions. Data from the hospitals were not included in this study, as our focus is on youth-serving institutions. Austin, located in central Texas, is a large capital city with over 960,000 residents within Austin and over 1 million residents when including surrounding cities [44]. All middle and high schools in the Austin Independent School District (n = 32) and 15 schools in other surrounding school districts were included in the Outdoor MEDIA Study. In order to be included in the present study, schools were required to have at least one advertisement within the prespecified radius that displayed a food or beverage item that could be coded for nutrient content including calories, fat (g), protein (g), carbohydrate (g), sugar (g), and sodium (mg). A total of 31 schools met this inclusion criterion. This study did not involve human or non-human animal subjects and was thus not required to obtain Institutional Review Board approval, as per posted guidance.

### 2.1. Data Collection

The research team created detailed maps with driving directions in order to systematically collect data along all streets within a half-mile radius of each middle and high school. A radius of a half-mile was chosen because it approximates the mean buffer size of similar studies published prior to data collection [8,21,22,23,42,45,46,47,48,49] and because youth who live within 800 m (~1/2 mile) of school are likely to walk to school [50,51] and thus likely to see displayed advertisements.

Using a detailed data collection protocol adapted from previous work [52,53] and a validated data collection tool [42], trained teams of data collectors documented all forms of outdoor marketing materials (e.g., advertisements attached to establishments, freestanding advertisements, posted menus, outlet logos) within the set radius [42]. This data collection tool, powered by FileMaker Pro (Filemaker PRO, Apple Inc. 2011, Cupertino, CA, USA) allowed data collectors to photograph and describe the advertisements on an iPod Touch while in the field and then perform additional, more detailed coding in the lab. Data collectors used detailed driving directions to systematically drive the half-mile radius around each school, stopping each time they observed a food or beverage advertisement or establishment. A food/beverage advertisement was defined as any sign promoting food or beverages (including logos and words). The data collectors took pictures of each advertisement, documented basic details about the advertisement (e.g., location, type), and noted the latitude and longitude.

Once data collection was complete, trained research assistants coded the data in the research lab for additional details and content. The coding protocol included nutrient content for advertisements containing an image of a food or beverage product.

Inter-rater reliability analyses were conducted to document the reliability of (1) documenting advertisements if they existed and (2) coding of the descriptive details of each advertisement. Seven schools were randomly selected, representing 12% of the total Outdoor MEDIA sample, including both urban and suburban schools. Two separate teams each completed a full data collection within 24 h of each other for all of the seven schools. Percent agreements were calculated for the overall tool reliability using a custom formula in FileMaker. Percent agreement was 70% (SE = 0.46) for documenting the presence of advertisements and 91.2% (SD = 0.02) for descriptive coding (e.g., coding the content of the advertisement). Detailed information on the data collection protocol and coding details can be found elsewhere [42,43].

### 2.2. Nutrient Content Coding

Only advertisements that contained an image of a food or beverage product that could be coded for nutrient content were included for nutrient content coding. Similar to previous research [39], nutrient content information was primarily gathered using the Nutrition Data System for Research (NDSR), United States Department of Agriculture (USDA), or through the product company website. Total energy (calories) and nutrient (fat, protein, carbohydrates, sugar, sodium) information was collected if available. If nutrient information for the advertised product was not available, the item was noted as unable to code for nutrient content. Unless product size was explicitly stated in the advertisement, we assumed a medium-sized portion.

### 2.3. School-Level Socioeconomic Status

Free or reduced-price lunch (FRPL) was used to measure school-level SES. To qualify for FRPL, a family must have household incomes at or below 130% or 185% of the Federal Poverty Line, respectively [54]. The Texas Education Agency (TEA) has publicly available FRPL data for their schools. For this study, using data from the TEA, schools with at least 60% of students receiving FRPL were classified as being located in areas with lower SES (n = 18), and those with less than 60% of students receiving FRPL were classified as being located in areas with higher SES (n = 13). The schools that were excluded for having zero advertisements that could be coded for nutrient content had on average 57.87% students receiving free or reduced-price lunch. Of the 16 excluded schools, 9 of them had FRPL > 60%.The 60% FRPL cut point has been used in previous research examining school-level income [55] and is the cut point used by the Food and Nutrition Service to determine if schools receive a lower or higher reimbursement for FRPL lunches served, where those schools with 60% or more FRPL qualification receive a slightly higher reimbursement than those schools with less than 60% FRPL [56].

The racial/ethnic composition of the included schools (reported in Table 1) was also collected from publicly available TEA data.

### 2.4. Statistical Analysis

The total number of advertisements displaying food and beverages that could be coded for nutrient content around each school and the total calories, fat (g), protein (g), carbohydrate (g), sugar (g), and sodium (mg) depicted in these advertisements were calculated. We performed a Shapiro–Wilk test of normality to investigate the distribution of each outcome, and in each case, the *p*-value was <0.05, which indicated that the null hypothesis of normality was rejected, and there was evidence that the data tested were not normally distributed. As such, we performed non-parametric Wilcoxon rank-sum tests, which are robust to data with skewness and kurtosis. Given the non-parametric statistics used and the relatively small sample size in terms of the number of schools in the analysis (n = 31), we performed a bootstrap procedure with 1000 repetitions to improve the precision of the models. Bootstrapping involves the random sampling of data with replacement from the original dataset, effectively simulating a larger sample size. We used bootstrapped Wilcoxon rank-sum tests to determine if there were significant differences between schools with lower SES and schools with higher SES on the of total advertisements and by nutrient content variables, averaged by school type. The racial/ethnic composition of the included schools was initially considered as a control variable; however, it was ultimately excluded from the analysis because racial/ethnic composition was highly collinear with FRLP (i.e., schools with high-% percentages of non-white students were also schools with high percentages of FRPL).

## 3. Results

A total of 31 schools were included in the present study. More than 32,000 students were enrolled in the included schools at the time of data collection. Information on the sociodemographic composition of the student body of included schools can be found in Table 1.

There were a total of 9132 unique advertisements collected. Of these, 3153 advertisements around the 31 schools displayed food and beverages that could be coded for nutrient content. Wilcoxon rank-sum tests of school-level characteristics of food and beverage advertising by SES of the schools are represented in Table 2. Schools with lower SES (≥60% of students receiving free or reduced-price lunch) had significantly more advertisements as compared to schools with higher SES, with 120 advertisements on average compared to 77 advertisements on average, respectively.

Advertisements around schools with lower SES contained products with significantly more total sodium (65,990 mg) compared to schools with higher SES (39,254 mg). There were no statistically significant differences between schools on caloric, fat (g), protein (g), carbohydrate (g), or sugar (g) content of the products displayed in the advertisements.

## 4. Discussion

Overall, this pilot study found that areas around schools where more than 60% of students qualify for FRPL have more food and beverage advertising and are exposed to significantly more total sodium than around schools where less than 60% of students qualify for FRPL. Specifically, we found that areas within 0.5 mile of middle and high schools with 60%+ of students receiving FRPL have more than 1.55 times greater food and beverage advertisements as compared to middle and high schools with less than 60% of students who receive FRPL (120 as compared to 77 respectively). The greater prevalence of food and beverage advertising indicates greater exposure to for children who attend these schools, suggesting that schools that serve communities with lower SES have substantially more advertisements than schools that serve communities with higher SES.

Our results are similar to the findings of other studies that show areas with limited income had significantly higher densities of food and beverage advertisements than higher income areas [7,8,9,41]. However, in contrast to Adams, Ganiti, and White [8], our study did find that schools located in areas of lower SES have advertisements with significantly higher levels of total sodium (more than 1.68 times greater) depicted compared to schools located in areas of higher SES. Our novel findings suggest that communities with lower incomes may not only be targeted with more advertisements but with advertisements for products that are nutritionally different than in communities with higher incomes. There are several possible reasons for the disparate findings between our study and Adams, Ganiti, and White’s, including geographical differences in advertising practices, as Adams, Ganiti, and White’s sample was from the UK, while our sample is from the US. Furthermore, our sample included nutrient content from a substantially larger sample of food and beverage advertisements—3153 advertisements in our study as compared to 211 advertisements in the Adams et al. study [8], which may have impacted our ability to observe statistically significant differences. Given the difference in findings between these studies, the only two to examine the nutrient content of outdoor food and beverage advertisements, more research is needed to draw stronger conclusions.

Our findings contribute to a growing body of literature examining the impact of the retail environment near schools on children’s diets. Much of the food children consume outside of the home is energy-dense and nutrient-poor [57,58], and research suggests that living in an unhealthy retail environments may be associated with children’s unhealthy food purchasing [59]. Our findings provide further insight into what the retail environments around schools look like. Future research can further examine if the nutritional composition of advertised food and beverage products have impact on students’ dietary behaviors.

The disparities in sodium content between schools with lower and higher SES parallel socioeconomic trends observed in sodium-related health conditions. There is ample evidence that increased sodium consumption is associated with higher blood pressure in children [60,61], which is a risk factor for cardiovascular disease [62,63], and that blood pressure tends to remain constant from childhood into adulthood [64,65]. There is also evidence that risk for hypertension is associated with SES [66,67] and evidence that children from lower SES backgrounds consume significantly more sodium than children from higher SES backgrounds [68]. Therefore, the finding that the areas around schools with lower SES were saturated with advertisements for products with significantly higher levels of sodium may contribute to our understanding of the socioeconomic associations with hypertension and cardiovascular disease. Additionally, the saturation of advertisements high in sodium may counter efforts to reduce sodium intake, as called for in the Healthy People 2030 objectives [69].

Our study has several strengths and limitations to note. This study is strengthened by the use of objectively collected data to document the food and beverage advertisement environment around schools and its focus on the content of the products within the advertisements. Additionally, our study coded every advertisement containing food or beverage items for specific nutrient information as opposed to broad categorizations into “healthy” or “unhealthy” products. This study provides nuanced, objective information about the outdoor food and beverage advertising environment that can be used to inform future research and policy efforts to regulate marketing. While there are notable strengths, there are also several weaknesses to note. First, this study had a relatively small sample of schools and was conducted in the Austin, Texas area, thus limiting generalizability. While the number of schools included in this study is small, the number of advertisements that were included far exceeds that of other studies with similar aims [8]. Additionally, while our use of objective nutrient content coding may be considered a strength, the healthfulness of a food or beverage cannot solely be determined by its nutrient content—this method does not allow us to examine whether products are nutritionally balanced. Another limitation is the use of free or reduced-price lunch as a proxy for school-level income. While FPRL is a commonly used metric for student- and school-level income, it is an imperfect measure of income, as it does not take into account income and expenses not encompassed in federal poverty line calculations [70]. Additionally, eligibility verification is imperfect, such that some students qualify/do not qualify in error [70]. However, FRPL data are collected consistently and are available for each school, making it accessible and comparable across schools. The age of the data used in this study is also a limitation—we cannot assume that the trends observed in this study have remained stagnant over time, and therefore, we cannot state that these findings reflect the current outdoor food marketing environment. Events such as the COVID-19 pandemic changed food-purchasing practices (e.g., increased food delivery and online ordering) as well as changes in the population of students attending public schools in person compared to before the pandemic—trends which the current study does not capture. While this certainly limits the ability of our findings to contribute to understanding the current food marketing environment, future research can leverage the age of our findings to understand how events like COVID-19 may have changed the outdoor food marketing environment near schools. Finally, this study is unable to address the directionality of influence in the relationship between advertising and consumption (i.e., do advertisements influence purchases or do purchases influence advertising).

Results from this pilot study can inform future research on the environmental influences of dietary behaviors, diet-related health disparities by income, and policy efforts to limit food and beverage advertising to youth. Future studies examining the nutrient contents of outdoor food and beverage advertising near schools might consider aiming to include a larger sample of schools, as a larger sample of schools may uncover associations beyond those presented in this study. Additionally, future studies might consider investigating the nutrient content of advertisements around schools in different geographic areas and at different times of year, as they may vary by region, urbanicity, or seasonality. Additionally, future work focused on specific types of product categories, such as fast food or sugar-sweetened beverages, can also help to determine how marketing and the nutritional content of marketing may vary by location. Beyond the nutrient content of advertisements, future research should examine how food and beverage products vary by community characteristics, as this would provide additional insight into the marketing practices of specific product industries. Furthermore, studies examining how outdoor advertisement exposure may impact dietary behaviors and diet-related health outcomes could provide further evidence for the role of marketing exposure on children’s health. Finally, in the data collection phase of this study, data collectors observed changes in advertisements within a 24 h period [42]. Longitudinal studies observing how outdoor advertisements may change over time are necessary to understand how to best study the outdoor food and beverage marketing environment. It is possible that the changes in outdoor food and beverage marketing environments may result in significantly different nutritional composition of products or remain relatively consistent. Until we understand how this environment changes, it is difficult to establish true exposure estimates.

Findings from this study have policy implications. While other jurisdictions have imposed restrictions on outdoor food and beverage advertising [71], these measures may not be possible at the federal level in the United States due to legal precedent [72]. However, some state and local governments have implemented—or are considering—policies to reduce youth marketing exposure, including regulations on the proximity of food outlets to schools [73]. Place-based policies, such as banning or restricting food and beverage advertisements near schools, would reduce youth exposure to unhealthy product marketing. This reduction would benefit all students but may have a greater impact on areas near schools that serve historically marginalized communities. For example, a simulation study found an outdoor tobacco advertising ban within 1000 feet of schools resulted in a significantly greater reduction of advertising near schools with a higher enrollment of Hispanic/Latino students [74]. Thus, policies aimed at regulating the outdoor marketing environment near schools may have a particular impact on areas with a higher density of advertisements. Additionally, industry self-regulation efforts, including the Children’s Food & Beverage Advertising Initiative (CFBAI), have not proven effective in reducing youth exposure to unhealthy products on television [75], though it likely reduced the total quantity of television food and beverage advertisements viewed by children [76]. Several prominent corporations have agreed to CFBAI regulations, including McDonalds, Coca-Cola, PepsiCo, Nestlé, and others [77]—companies that account for many of the advertisements included in this study. While CFBAI policies do not explicitly regulate outdoor advertising [77], CFBAI regulations may inadvertently affect signatory companies’ outdoor advertising practices. Currently, the CFBAI does not protect middle or high school students from unhealthy product marketing, as its primary demographic is children under 12 years of age [77]. Expanding self-regulation efforts to increase the age to 18 years old and under and to include outdoor advertising in their regulations would likely reduce the volume of advertisements and improve the nutritional content of the advertised products near middle and high schools. Efforts to reduce youth exposure to unhealthy food and beverage marketing could reduce the socioeconomic disparities observed in this study as well as the documented disparities in overweight and obesity prevalence. Efforts to reduce youth exposure to unhealthy food and beverage marketing could improve the diets of youth and, in turn, improve diet-related health outcomes.

## 5. Conclusions

Youth are exposed to environments oversaturated with advertisements for food products that are nutritionally poor. Specifically, this study provides evidence that the areas near schools with lower SES students are disproportionately targeted by food and beverage advertising, and are exposed to significantly more total sodium, when compared to the areas near schools with higher SES students. Additionally, our findings can inform policy endeavors aiming to regulate the outdoor marketing environment near schools. Our findings can inform future research examining the food and beverage marketing environment near schools as well as research investigating differences in the nutrient content of products by community economic resources.

## Figures and Tables

**Table 1:** Sociodemographic characteristics of higher-SES and lower-SES schools with food and beverage advertisements within one half mile of campus (n = 31).

	Students (#)	AAPI (%)	Black (%)	Hispanic (%)	White (%)	FRPL (%)
Higher SES (n = 13)	15,422	6.34	11.52	32.26	49.34	33.64
Lower SES (n = 18)	16,987	2.53	15.86	71.40	10.05	81.27

Note: AAPI = Asian ancestry or Pacific Islander; FRPL = free or reduced-price lunch.

**Table 2:** Wilcoxon rank-sum tests of school-level characteristics of Food and Beverage Advertising Around Schools with Higher and Lower SES in Austin, TX (n = 31).

Variable	Average Per-School	Range Per-School	Bootstrapped Z-Statistic	Bootstrapped Std. Error	*p*
Total Ads			2.01	0.88	0.044
Higher SES	77	1–517			
Lower SES	120	1–538			
Sum of Calories in Ads			1.69	0.97	0.091
Higher SES	37,409	1094–310,308			
Lower SES	43,935	1799–184,134			
Sum of Fat (g) in Ads			1.94	0.95	0.052
Higher SES	1743	20–16,112			
Lower SES	1504	75–4138			
Sum of Protein (g) in Ads			1.94	0.97	0.053
Higher SES	923	28–7156			
Lower SES	1242	47–4152			
Sum of Carbohydrates (g) in Ads			1.59	0.98	0.111
Higher SES	4708	106–34,501			
Lower SES	6589	206–34,337			
Sum of Sugar (g) in Ads			1.01	0.99	0.313
Higher SES	3312	17–24,904			
Lower SES	4316	18–26,910			
Sum of Sodium (mg) in Ads			2.20	0.89	0.028
Higher SES	39,254	1447–287,065			
Lower SES	65,990	2693–226,373			

Higher SES: Schools with <60% of students receiving free or reduced-price lunch (n = 13); Lower SES: Schools with ≥60% of students receiving free or reduced-price lunch (n = 18). School-level average values and ranges are rounded to the nearest whole number.

## Data Availability

The corresponding author can provide access to the dataset upon request.
